# Multi-drug resistant *Enterobacter bugandensis* species isolated from the International Space Station and comparative genomic analyses with human pathogenic strains

**DOI:** 10.1186/s12866-018-1325-2

**Published:** 2018-11-23

**Authors:** Nitin K. Singh, Daniela Bezdan, Aleksandra Checinska Sielaff, Kevin Wheeler, Christopher E. Mason, Kasthuri Venkateswaran

**Affiliations:** 1 Biotechnology and Planetary Protection Group, Jet Propulsion Laboratory, California Institute of Technology, M/S 89–2 4800 Oak Grove Dr, Pasadena, CA 91109 USA; 2 Department of Physiology and Biophysics, Weill Cornell Medicine, New York, NY USA; 3 Allosource, Centennial, CO USA; 4 The HRH Prince Alwaleed Bin Talal Bin Abdulaziz Alsaud Institute for Computational Biomedicine, Weill Cornell Medicine, New York, NY USA; 5 The Feil Family Brain and Mind Research Institute, Weill Cornell Medicine, New York, NY USA; 6 Present address: Washington State University (WSU) Extension – Youth and Families Program, WSU, Pullman, WA USA

**Keywords:** *Enterobacter*, International Space Station, Phylogenomic analyses

## Abstract

**Background:**

The antimicrobial resistance (AMR) phenotypic properties, multiple drug resistance (MDR) gene profiles, and genes related to potential virulence and pathogenic properties of five *Enterobacter bugandensis* strains isolated from the International Space Station (ISS) were carried out and compared with genomes of three clinical strains. Whole genome sequences of ISS strains were characterized using the hybrid de novo assembly of Nanopore and Illumina reads. In addition to traditional microbial taxonomic approaches, multilocus sequence typing (MLST) analysis was performed to classify the phylogenetic lineage. Agar diffusion discs assay was performed to test antibiotics susceptibility. The draft genomes after assembly and scaffolding were annotated with the Rapid Annotations using Subsystems Technology and RNAmmer servers for downstream analysis.

**Results:**

Molecular phylogeny and whole genome analysis of the ISS strains with all publicly available *Enterobacter* genomes revealed that ISS strains were *E. bugandensis* and similar to the type strain EB-247^T^ and two clinical isolates (153_ECLO and MBRL 1077). Comparative genomic analyses of all eight *E. bungandensis* strains showed, a total of 4733 genes were associated with carbohydrate metabolism (635 genes), amino acid and derivatives (496 genes), protein metabolism (291 genes), cofactors, vitamins, prosthetic groups, pigments (275 genes), membrane transport (247 genes), and RNA metabolism (239 genes). In addition, 112 genes identified in the ISS strains were involved in virulence, disease, and defense. Genes associated with resistance to antibiotics and toxic compounds, including the MDR tripartite system were also identified in the ISS strains. A multiple antibiotic resistance (MAR) locus or MAR operon encoding MarA, MarB, MarC, and MarR, which regulate more than 60 genes, including upregulation of drug efflux systems that have been reported in *Escherichia coli* K12, was also observed in the ISS strains.

**Conclusion:**

Given the MDR results for these ISS *Enterobacter* genomes and increased chance of pathogenicity (PathogenFinder algorithm with > 79% probability), these species pose important health considerations for future missions. Thorough genomic characterization of the strains isolated from ISS can help to understand the pathogenic potential, and inform future missions, but analyzing them in in-vivo systems is required to discern the influence of microgravity on their pathogenicity.

**Electronic supplementary material:**

The online version of this article (10.1186/s12866-018-1325-2) contains supplementary material, which is available to authorized users.

## Background

*Enterobacter* species are facultative anaerobic, Gram-stain-negative, and saprophytic microorganisms found in soil, sewage, and as a commensal enteric flora of the human gastrointestinal tract [1]. They have been associated with nosocomial infection in humans, causing bacteremia, endocarditis, septic arthritis, osteomyelitis, skin and soft tissue infections, lower respiratory tract, urinary tract, and intra-abdominal infections [2, 3]. Some *Enterobacter* have also been reported plant pathogens [4]. Antibiotic resistance and its clinical implications have been extensively studied in genus *Enterobacter*, especially *Enterobacter cloacae*, which is resistant to cephalosporins, ampicillin, amoxicillin, and cefoxitin [5, 6].

In an ongoing effort of the International Space Station (ISS) Microbial Observatory investigation, the National Aeronautics and Space Administration (NASA) is cataloging the total and viable microbial communities of crew-associated environments using cultivation and molecular techniques of microbial detection [7, 8]. As a result, five isolates belonging to the *Enterobacter bugandensis* group of bacteria from two different locations of the ISS were isolated [9]. Since the initial molecular screening identified these strains as *Enterobacter* but the identification was not able to resolve their taxonomy to species level, detailed genomic characterizations were warranted in addition to the traditional microbiological characterization. Due to its unstable taxonomic structure, methods utilized for the speciation of *Enterobacter* varied widely. Commercial biochemical typing systems such as API® 20E [10] or Vitek® 2, and matrix-assisted laser desorption ionization–time of flight mass spectrometry (MALDI-TOF MS) [11] methods have been used, but with limited success. On the basis of 16S rRNA analysis, *Enterobacter* was structured as a polyphyletic genus and most of the species could not be resolved [1]. Therefore, multilocus sequence typing (MLST) analysis was found to be more appropriate for phylogenetic classification of *Enterobacter* species [12].

To resolve this question further, whole genome sequencing (WGS) and de novo assembly was performed on all five ISS *E. bugandensis* strains, creating MLST and genome variation profiles of the ISS strains [13]. Furthermore, comparative genome alignment of the ISS strains with all publicly available 1291 *Enterobacter* genomes revealed that genomes of these five ISS strains were highly similar to only three clinical *E. bugandensis* with very high genome similarities and formed a unique ecotype. They are (a) EB-247 strain [13], isolated from neonatal blood of a patient from Tanzania, (b) 153_ECLO strain [14], isolated from the urine of a neonatal patient strain admitted to the University of Washington Medical Center, Seattle, WA and (c) MBRL 1077 strain, a carbapenemase-producing strain [15] isolated from the wound of a 72-year-old woman with a history of cutaneous scleroderma, medically complicated obesity, and venous insufficiency. In this study, comparative genomic analyses of five ISS strains and three clinical isolates were carried out to elucidate antimicrobial resistance (AMR) phenotypic properties, MDR gene profiles, and genes related to potential virulence and pathogenic potential of the ISS *Enterobacter* strains.

## Methods

Sample collection from ISS environmental surfaces, processing, cultivation of bacteria were already reported [9]. When 105 bacterial strains isolated from various ISS locations were analyzed for their phylogenetic affiliations, five isolates were identified as *Enterobacter bugandensis*. The five *Enterobacter* isolates characterized during this study were isolated from two different locations of the ISS flight in March 2015. Four isolates were isolated from the waste and hygiene compartment (WHC), and one strain from the Advanced Resistive Exercise Device (ARED) foot platform of ISS.

### Phenotypic characterization

The isolates were biochemically identified using Vitek®2 Compact gram-negative (GN) cards (bioMerieux, Inc., Hazelwood, MO) [16] and BioLog (Hayward, CA) carbon substrate utilization profile characterization [17]. Sample preparation for MALDI-TOF MS protein analysis was carried out as previously established [18]. MALDI-TOF mass spectra were obtained from an Ultraflex III instrument (Bruker Daltonik, Billerica, MA) operated in linear positive mode under Flex-Control 3.1 software. Mass spectra were processed using Flex Analysis (version 3.1; Bruker Daltonik) and BioTyper software (version 3.1; Bruker Daltonik).

### Genome sequence analysis

Genomic DNA extraction was performed as described previously [9]. WGS was performed on the Oxford Nanopore MinION (Oxford, United Kingdom) and Illumina MiSeq sequencing platform (San Diego, CA). A hybrid approach was utilized for genome assembly using reads from both platforms. Nanopore reads were processed using Poretools [19] toolkit for the purposes of quality control and downstream analysis. Error corrected Nanopore and MiSeq reads were assembled using SPAdes [20]. Scaffolding of the assembled contigs was done using SSpace [21] and gap filling was executed using GapFiller [22]. The draft genomes after assembly and scaffolding were annotated with the help of the Rapid Annotations using Subsystems Technology (RAST) [23] and RNAmmer servers [24] for downstream analysis [25, 26] ISS strains assembly characteristics are given in Additional file 1: Table S1. The 16S rRNA, *gyrB,* and *rpoB* gene sequences were retrieved from the WGS and analyzed for their phylogenetic affiliations. The neighbor-joining phylogenetic analysis was performed using the MEGA7 software package [27]. MLST analysis was carried out as described previously [28]. The MLST scheme employed here uses seven house-keeping genes: *dnaA* (DNA replication initiator), *fusA* (codes Elongation factor G), *gyrB* (DNA replication and repair), *leuS* (Leucine tRNA ligase), *pyrG* (CTP synthase), *rplB* (50S ribosomal protein), and *rpoB* (β subunit of bacterial RNA polymerase) [29]. The retrieved sequences were compared with the sequence types deposited at *E. cloacae* MLST database [30], concatenated according to the MLST scheme. The genes were analyzed independently, or as a single concatenate using neighbor-joining algorithms.

The SNP-based phylogenetic tree was generated using CSIPhylogeny [28] version 1.4. Using genome sequences of multiple isolates CSIPhylogeny calls SNP, filters the SNPs, performs site validation, and infers a phylogeny based on the concatenated alignment of high-quality SNPs. The analysis included *Enterobacter* reference whole genome sequences which were downloaded from the NCBI GenBank database. This genome-wide SNP analysis allows for higher resolution phylogenetic analysis compared to other methods, which is necessary for comparing highly similar genomes. All positions containing gaps and missing data were eliminated. A total of 3832 positions in the dataset were used to confer the final tree.

Hybrid-genome-assembly (ONT and Illumina data) of strain IF3SW-P2 was nominated as reference genome of the 5 strains sequenced. The IF3SW-P2 genome was used to realign the Illumina MiSeq reads with reads of other 4 strains using bwa-mem (http://bio-bwa.sourceforge.net/). Postprocessing of the BAM files was performed using SAMtools [31] and picard (https://github.com/broadinstitute/picard). GATK HaplotypeCaller (https://software.broadinstitute.org/gatk/) was used for SNP and indels identification.

Pairwise average nucleotide index (ANI) was calculated using the algorithm from Goris et al. 2007 [32] and GC content was determined using EzTaxon-e [33]. Digital DNA-DNA hybridization (dDDH) was performed using the Genome-to-Genome Distance Calculator 2.0 (GGDC 2.0) [34]. Briefly, the genome sequences in FASTA format were submitted to GGDC 2.0 along with the sequences in FASTA format for the *Enterobacter* reference genome that were available: *E. aerogenes* KCTC 2190, *E. asburiae* ATCC 35953, *E. bugandensis* EB-247^T^, *E. cancerogenus* ATCC 35316, *E. cloacae* ATCC 13047, *E. hormaechei* ATCC 49162, *E. kobei* DSM 13645, *E. lignolyticus* SCF1, *E. ludwigii* EN119, *E. massiliensis* JC163, *E. mori* LMG25706, *E. muelleri* JM-458^T^, *E. xiangfangensis* LMG 27195, and *E. soli* ATCC BAA-2102*.* The results were obtained by comparing query genomes (ISS isolates) with each of the reference genomes to calculate dDDH and intergenomic distances. Global comparison of ISS isolates with other species was done using local BLAST [35]. Genome sequence assemblies were aligned using BLASTN and the diagrammatic view was created using BLAST Ring Image Generator (BRIG) software [36].

### Nucleotide sequence deposition

The WGS data submitted to the National Center for Biotechnology Information (NCBI) GenBank and NASA GenLab databases were downloaded and characterized during this study. The complete genome sequences of all ISS strains were deposited in NCBI under Bioproject PRJNA319366 as well as at the NASA GeneLab system (GLDS-67; https://genelab-data.ndc.nasa.gov/genelab/accession/GLDS-67/#). The GenBank/EMBL/DDBJ accession numbers for the 16S rRNA gene sequence of isolated strains are: IF2SW-B1 (KY218809), IF2SW-B5 (KY218813), IF2SW-P2 ^T^ (KY218815), IF2SW-P3 (KY218816), and IF3SW-P2 (KY218819).

## Results

### Phenotypic characteristics

The ISS strains showed aerobic, motile, rod shape, Gram stain negative characteristics; colonies were pale yellow in color, formed within 24–36 h at 35 °C on R2A, TSA, and blood agar. Growth was observed at 1–8% NaCl and in pH range 5–7. The Vitek and BioLog systems as well as MALDI-TOF profiles identified the ISS strains as *E. ludwigii.* The MALDI-TOF profile scores for the tested strains were 2.16 *(E. ludwigii*) and 2.10 (*E. asburiae*)*.* In general, no noticeable phenotypic differences were observed among the *Enterobacter* species tested including *E. bugandensis* EB*-*247^T^, whose genome is closer to ISS strains. As reported earlier, all these five ISS *Enterobacter* isolates were resistant to cefazolin, cefoxitin, oxacillin, penicillin and rifampin, while for ciprofloxacin and erythromycin, strains were either resistant or intermediate resistant. For gentamycin and tobramycin some strains were resistant, some intermediate resistant, and some susceptible [9].

### Molecular phylogeny

The 16S rRNA gene sequencing of all five isolates placed them within the *Enterobacter* group and showed maximum similarity (99.6%) with *E. bugandensis* EB-247^T^, *E. cancerogenus* LMG 2693, *E. ludwigii* EN-119, and *E. mori* R18–2 (99 to 100%). Since 16S rRNA gene sequencing analysis is insufficient to differentiate *Enterobacter* species, polygenic and whole genome-based analyses were further attempted. All ISS strains were phylogenetically characterized by the *gyrB* locus (~ 1.9 kb) and showed that the ISS isolates form a close group with *E. bugandensis* EB-247^T^ and 153_ECLO strains (> 99%) while MBRL 1077 isolate was exhibiting 97% similarity with high bootstrap value.

### MLST analysis

**Fig. 1:**
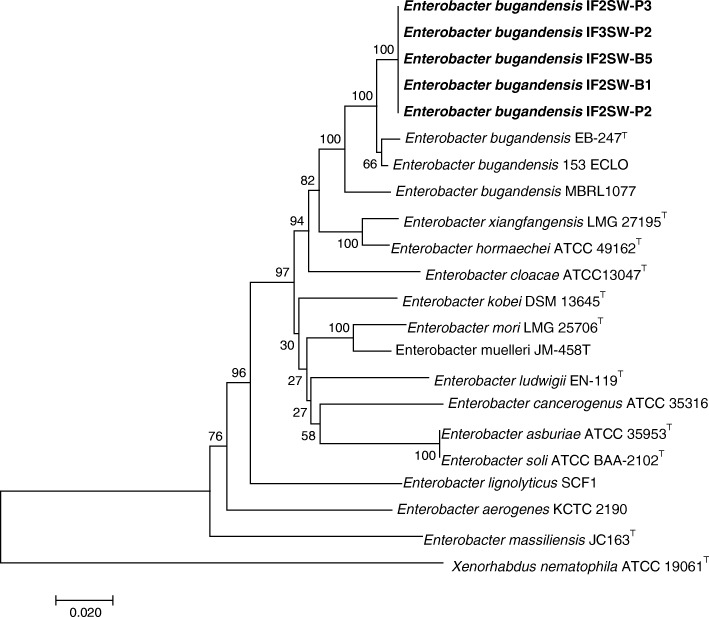
Multiple-locus sequence types (MLST) analysis of ISS strains and related species of the *Enterobacter*. The obtained genomic contigs of the ISS isolates (in bold) were searched for gene sequences of *dnaA*, *fusA*, g*yrB*, *leuS*, *pyrG*, *rplB*, and *rpoB*, which are standardized for the use in MLST analysis and reported for *E. cloacae* species [29]. The retrieved sequences were compared with the sequence types deposited at the *Enterobacter* MLST database, concatenated according to the MLST scheme. The reconstruction was based on the RAxML algorithm [4], and the bootstrap values were calculated using 1000 replicates. The bar indicates 2% sequence divergence

The genomic contigs of the ISS isolates were searched for gene sequences of *dnaA, fusA, gyrB. leuS, pyrG, rplB, and rpoB*, which are standardized for the use of MLST analysis and reported for *E. cloacae* species [29]. The good congruence between the single-gene reconstructions and the concatenate reinforced the stability of the genealogy were observed. The reconstruction was based on the RAxML algorithm [37] and the resulting MLST tree (Fig. 1) shows that the ISS isolates are phlylogenetically related to *E. bugandensis* clinical strains (EB-247, strain 153_ECLO, and isolate MBRL 1077).

### SNP analysis

**Fig. 2:**
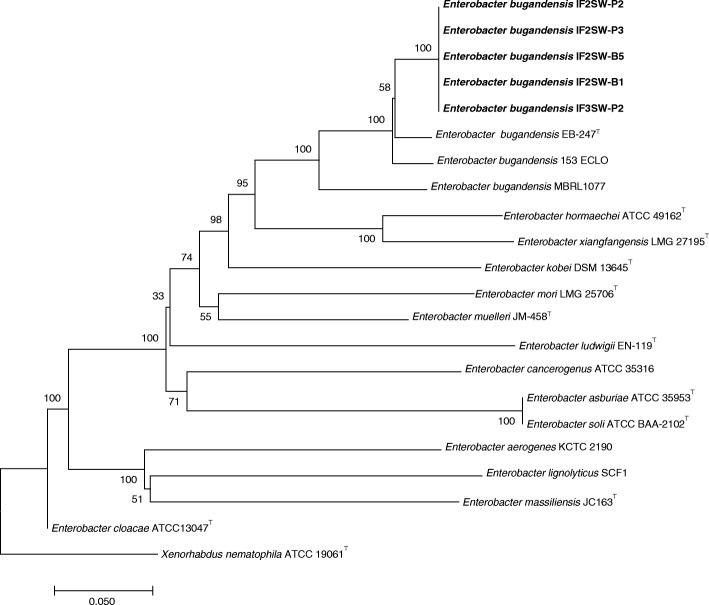
Single nucleotide polymorphism (SNP) based phylogenetic tree, showing the relationship between the ISS isolates (in bold) and members of the *Enterobacter* genus. The tree was generated using CSI Phylogeny [28] version 1.4

Even though MLST analysis was clearly able to genomically resolve the ISS isolates to species level and distinguish them from other members of the genus *Enterobacter*, whole genome SNP analysis, SNP tree analysis excluding plasmid sequences, was carried out to validate these results. The snpTree does not ignore any nucleotide positions and is able to consider 100% of the chromosomal genome. All the available WGS of the *Enterobacter* genus reference genomes from GenBank were used for SNP analysis with snpTree. Of the 22 total nucleotide sequences; 58,121 positions were found in all analyzed genomes and 3832 positions in the dataset were used to confer the final tree (Fig. 2). The snpTree analyses confirmed and gave a strong validation to the MLST/*gyrB* data, confirming that all ISS isolates are *E. bugandensis* but strain MBRL 1077 grouped differently from the members of the *E. bugandensis* group*.*

SNP identification within ISS strains was carried out using GATK HaplotypeCaller. Filtered SNP calls and indels (after removal of false positives) are given in the Additional file 1: Table S1. Post-filtration analyses showed that there were 9, 12, 15, 13, and 0 SNPs seen in IF2SWB1, IF2SWB5, IF2SWP2, IS2WP3 and IS3SWP2, respectively. Further 6, 0, 4, 6, and 0 indels were seen in IF2SWB1, IF2SWB5, IF2SWP2, IS2WP3 and IS3SWP2, respectively (Additional file 1: Table S1). A maximum of 15 SNPs was observed among ISS isolates, probably being clonal in origin, with a very recent common ancestor. However, it should be noted that 4 strains were isolated from location #2 (space toilet) and one strain from the exercise platform (ARED).

### ANI values and digital DNA-DNA hybridization

The ANI values for the ISS strains were maximum against *E. bugandensis* EB-247, 153_ECLO, and MBRL 1077 strains (> 95%) as were those of MLST analyses, and the ANI values of rest of the *Enterobacter* genomes tested were < 91% (Table 1). The digital DNA-DNA hybridization (dDDH) results of the ISS strain showed high similarity with *E. bugandensis* EB-247 (89.2%), 153_ECLO (89.4%), and MBRL 1077 (64%) strains whereas dDDH value was < 44.6% to all the other available *Enterobacter* reference genomes (Table 1). Based on various molecular analyses attempted during this study all five ISS *Enterobacter* strains were phenotypically and genotypically identified as *E. bugandensis.*

**Table 1:** Digital DDH and ANI values of ISS strains and comparison with various *Enterobacter* species

Bacteria	Strain number	Source	GenBank accession number	ISS *Enterobacter bugandensis* isolates (*n* = 5)	
				dDDH	ANI (%)
*E. bugandensis*	IF2SW-P2	ISS-WHC	POUR00000000	100	100.00
*E. bugandensis*	IF2SW-B1	ISS-WHC	POUQ00000000	100	99.99
*E. bugandensis*	IF2SW-B5	ISS-WHC	RBVJ00000000	100	99.99
*E. bugandensis*	IF2SW-P3	ISS-WHC	POUP00000000	100	99.99
*E. bugandensis*	IF3SW-P2	ISS-AREED	POUO00000000	100	99.99
*E. bugandensis*	EB-247^T^	Nosocomial	FYBI00000000	89.2	98.66
*E. bugandensis*	153 ECLO	Nosocomial	NZ_JVSD00000000	89.4	98.73
*E. bugandensis*	MBRL1077	Nosocomial	PRJNA310238	63.9	95.26
*E. aerogenes*	KCTC 2190	Nosocomial	CP002824	22.7	78.74
*E. asburiae*	ATCC 35953^T^	Nosocomial	NZ_CP011863	30.4	85.59
*E. cancerogenus*	ATCC 35316	Stool	NZ_ABWM00000000	31.8	86.10
*E. cloacae*	ATCC 13047^T^	Spinal fluid	NC_014121	35.4	87.91
*E. hormaechei*	ATCC 49162^T^	Sputum	AFHR01000000	35.4	87.82
*E. kobei*	DSM 13645^T^	Blood	NZ_CP017181	42.8	90.54
*E. lignolyticus*	SCF1	Soil	CP002272	23.5	79.98
*E. ludwigii*	EN-119^T^	Human	NZ_CP017279	34.4	87.57
*E. massiliensis*	JC163^T^	Stool	NZ_CAEO00000000	22.8	79.07
*E. mori*	LMG 25706^T^	Mulberry	NZ_AEXB00000000	37.0	88.59
*E. muelleri*	JM-458^T^	Rhizosphere	FXLQ00000000	44.6	90.77
*Xenorhabdus nematophila*	ATCC 19061^T^	Intestine	FN667742	22.8	69.41

*dDDH* digital DNA-DNA hybridization, *ANI* Average Nucleotide Identity, *WHC* Waste and Hygiene Compartment, *ARED* Advanced resistive exercise device (ARED) foot platform

### Functional characteristics

**Fig. 3:**
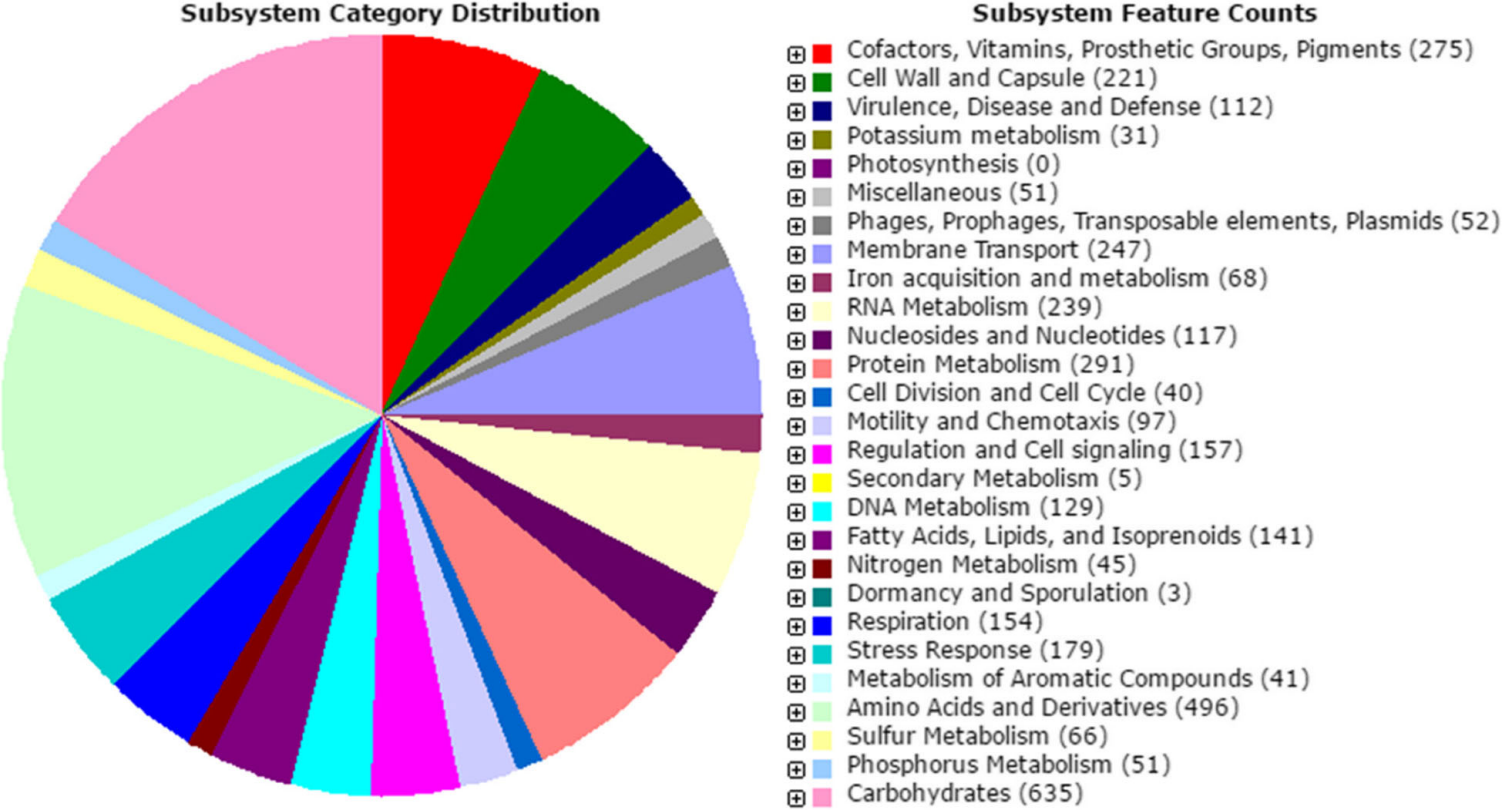
Metabolic functional profiles and subsystem categories distribution of strain IF3SW-P2. 4733 genes were identified that dominated by carbohydrate metabolism followed by amino acid and derivatives

A detailed genome analysis of all five ISS strains and 3 clinical isolates were carried out to understand its genetic makeup. A total of 4733 genes were classified as carbohydrate metabolism (635 genes), amino acid and derivatives (496 genes), protein metabolism (291 genes), cofactors, vitamins, prosthetic groups, pigments (275 genes), membrane transport (247 genes), and RNA metabolism (239 genes) (Fig. 3). To test antimicrobial resistance at genomic level, the ISS strains were further compared with nosocomial isolates (1291 genomes) having more than 95% ANI identity with the ISS strains, which taxonomically identified them as same species. Genomes of the clinical strains of *E. bugandensis* 247, 153_ECLO, and MBRL-1077, whose ANI values were > 95%, were used for the genetic comparison to further broaden the picture.

Features playing a broad role and implemented by the same domain such as Spectinomycin 9-O-adenylyltransferase and Streptomycin 3-O-adenylyltransferase (EC 2.7.7.47) were only present in *E. bugandensis* 247 due to the probable lack of selective pressure that might have been encountered by the ISS isolates (Table 2). The predicted arsenic resistance (arsenic resistance protein, ArsH) noticed in *E. bugandensis* 247 but not in other strains should be phenotypically tested to confirm the resistance properties conferred in strain *E. bugandensis* 247 and cross checked with the ISS strains for their inability to degrade arsenic. Trace metals detected in ISS potable water samples, but typically below potability requirements, included arsenic, barium, chromium, copper, iron, manganese, molybdenum, nickel, lead, selenium, and zinc. No mercury or cadmium was detected and the arsenic levels varied from nondetectable in water samples to a maximum of 3.8 μg/L [38].

**Table 2:** Comparative analyses of antimicrobial gene profiles of *E. bungandensis* isolated from ISS and clinical sources

AMR genes and its role	AMR genes that are present in the strains that are:			
	ISS (n = 5)	153 ECLO	MBRL 1077	EB247
Cystine ABC transporter, ATP-binding protein	+	+	+	+
Cystine ABC transporter, permease protein	+	+	+	+
D-cysteine desulfhydrase (EC 4.4.1.15)	+	+	+	+
Spectinomycin 9-O-adenylyltransferase				+
Streptomycin 3-O-adenylyltransferase (EC 2.7.7.47)				+
Arsenate reductase (EC 1.20.4.1)	+	+	+	+
Arsenic efflux pump protein	+	+	+	+
Arsenic resistance protein ArsH				+
Arsenical resistance operon repressor	+	+	+	+
Beta-lactamase (EC 3.5.2.6)	+	+	+	+
Beta-lactamase class C and other penicillin binding proteins			+	
Metal-dependent hydrolases of the beta-lactamase superfamily I	+	+	+	+
Cation efflux system protein CusA	+	+	+	+
Cation efflux system protein CusC precursor			+	+
Cation efflux system protein CusF precursor			+	+
Cobalt-zinc-cadmium resistance protein	+	+	+	+
Cobalt-zinc-cadmium resistance protein CzcA	+	+	+	+
Cobalt/zinc/cadmium efflux RND transporter, membrane fusion protein, CzcB family			+	+
Copper-sensing two-component system response regulator CusR			+	+
DNA-binding heavy metal response regulator	+	+	+	+
Heavy metal sensor histidine kinase				+
Probable Co/Zn/Cd efflux system membrane fusion protein	+	+	+	+
Zinc transporter ZitB	+	+	+	+
Acetyl-coenzyme A carboxyl transferase beta chain (EC 6.4.1.2)	+	+	+	+
Amidophosphoribosyltransferase (EC 2.4.2.14)	+	+	+	+
Colicin V production protein	+	+	+	+
DedA protein	+	+	+	+
DedD protein	+	+	+	+
Dihydrofolate synthase (EC 6.3.2.12)	+	+	+	+
Folylpolyglutamate synthase (EC 6.3.2.17)	+	+	+	+
tRNA pseudouridine synthase A (EC 4.2.1.70)	+	+	+	+
Blue copper oxidase CueO precursor	+	+	+	+
Copper resistance protein C precursor	+	+	+	+
Copper resistance protein D	+	+	+	+
Copper-translocating P-type ATPase (EC 3.6.3.4)	+	+	+	+
Copper homeostasis protein CutE	+	+	+	+
Copper homeostasis protein CutF precursor	+	+	+	+
Magnesium and cobalt efflux protein CorC	+	+	+	+
Membrane protein, suppressor for copper-sensitivity ScsB	+	+	+	+
Membrane protein, suppressor for copper-sensitivity ScsD	+	+	+	+
Secreted protein, suppressor for copper-sensitivity ScsC	+	+	+	+
Suppression of copper sensitivity: putative copper binding protein ScsA	+	+	+	+
Fosfomycin resistance protein FosA	+	+	+	+
Membrane-bound lysozyme inhibitor of c-type lysozyme	+	+	+	+
16 kDa heat shock protein A	+	+	+	+
16 kDa heat shock protein B	+	+	+	+
HTH-type transcriptional regulator YidP	+	+	+	+
Mediator of hyperadherence YidE	+	+	+	+
Outer membrane lipoprotein YidQ	+	+	+	+
Uncharacterized protein YidR	+	+	+	+
Mercuric ion reductase (EC 1.16.1.1)				+
PF00070 family, FAD-dependent NAD(P)-disulphide oxidoreductase	+	+	+	+
Mercuric resistance operon coregulator				+
Mercuric resistance operon regulatory protein				+
Mercuric transport protein, MerE				+
Acriflavin resistance protein	+	+	+	+
Macrolide export ATP-binding/permease protein MacB (EC 3.6.3.-)	+	+	+	+
Macrolide-specific efflux protein MacA	+	+	+	+
Membrane fusion protein of RND family multidrug efflux pump	+	+	+	+
Multi antimicrobial extrusion protein (Na(+)/drug antiporter), MATE family of MDR efflux pumps	+	+	+	+
Multidrug-efflux transporter, major facilitator superfamily (MFS) (TC 2.A.1)	+	+	+	+
Probable transcription regulator protein of MDR efflux pump cluster	+	+	+	+
RND efflux system, inner membrane transporter CmeB	+	+	+	+
RND efflux system, membrane fusion protein CmeA	+	+	+	+
RND efflux system, outer membrane lipoprotein CmeC	+	+		+
RND efflux system, outer membrane lipoprotein, NodT family	+	+	+	+
Transcription repressor of multidrug efflux pump acrAB operon, TetR (AcrR) family	+	+	+	+
Type I secretion outer membrane protein, TolC precursor	+	+	+	+
Inner membrane component of tripartite multidrug resistance system	+	+	+	+
Membrane fusion component of tripartite multidrug resistance system	+	+	+	+
Outer membrane component of tripartite multidrug resistance system	+	+	+	+
Multiple antibiotic resistance protein MarA	+	+	+	+
Multiple antibiotic resistance protein MarB	+	+	+	+
Multiple antibiotic resistance protein MarC	+	+	+	+
Multiple antibiotic resistance protein MarR	+	+	+	+
DNA-directed RNA polymerase beta subunit (EC 2.7.7.6)	+	+	+	+
DNA-directed RNA polymerase beta' subunit (EC 2.7.7.6)	+	+	+	+
LSU ribosomal protein L20p	+	+	+	+
LSU ribosomal protein L35p	+	+	+	+
Translation initiation factor 3	+	+	+	+
SSU ribosomal protein S12p (S23e)	+	+	+	
SSU ribosomal protein S7p (S5e)	+	+	+	
Translation elongation factor G	+	+	+	
Translation elongation factor Tu	+	+	+	
L-aspartate oxidase (EC 1.4.3.16)	+	+	+	+
Quinolinate phosphoribosyltransferase [decarboxylating] (EC 2.4.2.19)	+	+	+	+
Quinolinate synthetase (EC 2.5.1.72)	+	+	+	+
DNA gyrase subunit A (EC 5.99.1.3)	+	+	+	+
DNA gyrase subunit B (EC 5.99.1.3)	+	+	+	+
Topoisomerase IV subunit A (EC 5.99.1.-)	+	+	+	+
Topoisomerase IV subunit B (EC 5.99.1.-)	+	+	+	+
Streptothricin acetyltransferase, Streptomyces lavendulae type	+	+	+	+
Multidrug transporter MdtB	+	+	+	+
Multidrug transporter MdtC	+	+	+	+
Multidrug transporter MdtD	+	+	+	+
Probable RND efflux membrane fusion protein	+	+	+	+
Response regulator BaeR	+	+	+	+
Sensory histidine kinase BaeS	+	+	+	+
Conserved uncharacterized protein CreA	+	+	+	+
Inner membrane protein CreD	+	+		
Two-component response regulator CreB	+	+		
Two-component response regulator CreC	+	+		

### Global comparison of ISS genomes with other ***Enterobacter*** genomes

**Fig. 4:**
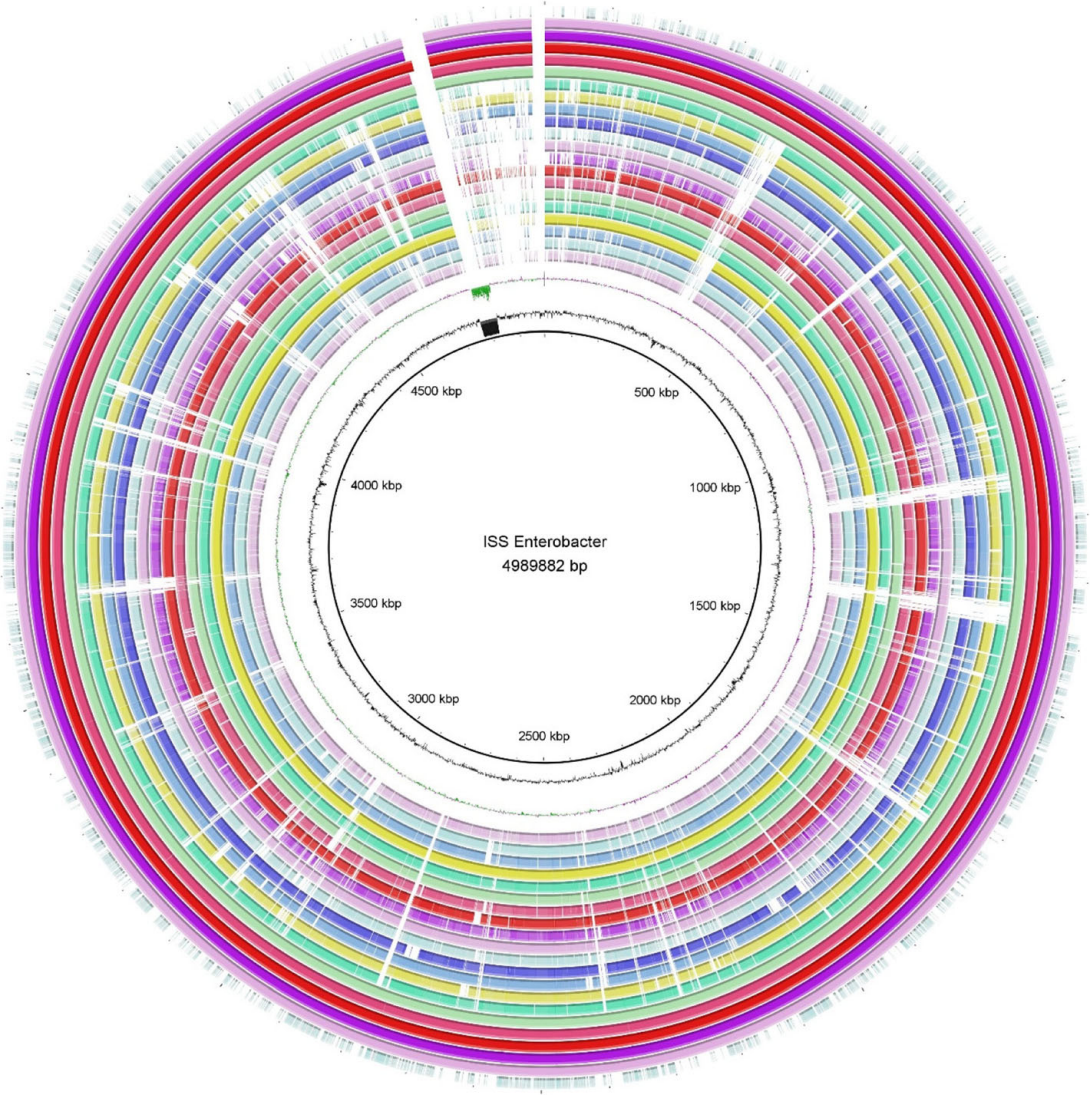
Global comparison of ISS *E. bugandensis* with other *Enterobacter* WGS from NCBI Microbial Genomes Resource was done using BRIG. Genome sequence assemblies were aligned using BLASTN and the diagrammatic view was created using BRIG software. The innermost ring indicates the genomic position of the reference genome (*E. bugandensis 247*^*T*^), next ring indicates GC content, and the third ring indicates GC skewness. The remaining 21 rings indicate the presence or absence of BLASTN hits at that position. Each ring represents WGS of single *Enterobacter* species, each shown in different color. Positions covered by BLASTN alignments are indicated in solid colors and gaps (white spaces) represent genomic regions not covered by BLASTN alignments. Order of genome from inner ring to outer is as follow: *E. aerogenes* KCTC 2190, *E. asburiae* ATCC 35953 T, *E. bugandensis* EB-247^T^, *E. cancerogenus* ATCC 35316, *E. bugandensis* 153_ECLO, *E. cloacae* ATCC 13047^T^, *E. bugandensis* MBRL1077, *E. hormaechei* ATCC 49162^T^, *E. kobei* DSM 13645^T^, *E. lignolyticus* SCF1, *E. ludwigii* EN-119^T^, *E. massiliensis* JC163^T^, *E. mori* LMG 25706^T^, *E. muelleri* JM-458^T^, *Enterobacter soli* ATCC BAA-2102^T^, *Enterobacter xiangfangensis* LMG 27195^T^, *E. bugandensis* IF2SW-B1, *E. bugandensis* IF2SW-B5, *E. bugandensis* IF2SW-P2, *E. bugandensis* IF2SW-P3, *E. bugandensis* IF3SW-P2, *Xenorhabdus nematophila* ATCC 19061^T^

A visualization program was reported to be invaluable [36] in determining the genotypic differences between closely related prokaryotes. Visualizing a prokaryote genome as a circular image has become a powerful means of displaying informative comparisons of one genome to a number of others. Using BRIG, a global visual comparison of ISS isolates with other *Enterobacter* WGS from the GenBank Microbial Genomes Resource was carried out. The resulting output of the BRIG analysis [36], a visualization image, showed draft genome assembly information, read coverage, assembly breakpoints, and collapsed repeats. The mapping of unassembled sequencing reads of the ISS genomes against fully annotated *E. cloacae* central reference sequences is depicted in Fig. 4.

## Discussion

In summary, a comparative phenotypic and genotypic analyses of ISS isolates identified as *E. bugandensis* were carried out*.* Additional genomic analyses revealed a close genetic relatedness between ISS isolates and nosocomial earth isolates. MLST and whole genome SNP tree placed ISS and nosocomial isolates to a separate clade when phylogenetically aligned with other member of genus *Enterobacter*. A detailed functional and antimicrobial resistance analysis reveals that the ISS isolates have a 79% probability of being a human pathogen and share similar antimicrobial resistance pattern with *E. bugandensis* EB-247*,* MBRL-1077 and 153_ECLO strains, making them relevant for future missions and crew health considerations.

A total of 112 identified genes of the ISS strains were involved in virulence, disease, and defense. Genes associated with resistance to antibiotics and toxic compounds, including the multidrug resistance tripartite system (also known as 3-protein systems) as shown in a polychlorinated biphenyl-degrader, *Burkholderia xenovorans* LB400 [39], was noticed in the ISS strain. This protein forms the basic structure and plays a crucial role in, the functioning of an efflux pump rendering a microbe drug resistant [40, 41]. A multiple antibiotic resistance (MAR) locus or MAR operon was observed in ISS strains, which codes for protein MarA, MarB, MarC, and MarR, and regulate more than 60 genes, including upregulation of drug efflux systems that have been reported in *Escherichia coli* K12 [42–44]. Aminoglycoside adenylyltransferases, whose role is spectinomycin 9-O-adenylyltransferases, which confers microbial resistance to the aminoglycosides in *Salmonella enterica,* was also seen in ISS strains [45]. Similarly, resistance to fluoroquinolones due to a mutation in *gyrA* gene in *S. enterica* [46], and fosfomycin resistance due to the presence of FosA protein-coding gene, which catalyzes the addition of glutathione to C1 of the oxirane in *Serratia marcescens* [47], were observed in ISS strains. Multiple copies of multi-drug resistance (MDR) genes highly homologous to *S. marcescens*, a pathogen, were identified in the ISS *Enterobacter* genomes, which gives an indication that these strains may be a potential human pathogen. When tested with PathogenFinder [48] algorithm, strain IF2SW-P2^T^ had > 77% probability to be a human pathogen. When compared with *E. cloacae* ATCC 13047, which is a well-described human pathogen [49], all five ISS strains showed a > 79% probability score.

Astronauts have been taking beta-lactam based medical drugs for approximately two decades, and ß-lactamase (superfamily I [metal dependent hydrolases] and E.C.3.5.2.6) was present in all strains under study, while penicillin-binding proteins (PPB4B) were only present in MBRL-1077. Fluoroquinolone resistance due to gyrase and topoisomerase mutation was present in all the strains. Metal-dependent hydrolases, cation efflux system protein CusA, cobalt-zinc-cadmium resistance protein, cobalt-zinc-cadmium resistance protein CzcA, DNA-binding heavy metal response regulator, Co/Zn/Cd efflux system membrane fusion protein, zinc transporter ZitB were found in both ISS isolate and nosocomial organism understudy. These genes principally help in detoxification of periplasm by exporting toxic metal cation outside the cell. Determinants of the metal resistance are usually located on the plasmid and readily acquired from the environment and also complement antibiotic resistance [50, 51]. The plasmid encoded putative transcriptional regulators containing the CopG/Arc/MetJ DNA-binding domain and a metal-binding domain were present in the ISS strains (Additional file 2: Table S2). Further studies are required for phenotypic characterization to confirm this trait. Presence of active beta lactamase gene, efflux pump, and RND (resistance, nodulation and cell division protein family) protein family renders broad-spectrum resistance to ISS isolates from drugs and natural inhibitors.

We have recently observed that competency of bacteria to acquire foreign genetic material increases in microgravity (in preparation) and similar mechanism for metal resistance of ISS strain was also predicted. Antimicrobial and metal resistance is also conferred by RND genes [52], which were present in all the strains under study. Genomic analysis reveals the presence of genes associated with MDR efflux pump, belonging to RND, which are reported to be the major contributors of resistance to antibiotic and other toxic compounds to the bacteria [41]. RND efflux system, inner membrane transporter CmeB, membrane fusion protein CmeA, outer membrane lipoprotein CmeC, outer membrane lipoprotein NodT family were found in all strains. These become important for the future space studies, as MDR has been reported to play role in the physiological function and confer resistance to the substances like bile, hormone and host defense molecule [53], which can make bacteria a dominant persistor and lead to pathogenicity in humans.

## Conclusion

The genomic characterizations showed that the ISS *Enterobacter* strains might potentially exhibit pathogenicity to human. However, the pathogenicity of the ISS strains compared to clinical strains isolated from patients should be explored in vivo experiments before making any assumption about whether these potential AMR gene markers are due to spaceflight changes or not. Moreover, the transit time and route for the organisms from the ISS may have some small impact on the response or physiological traits of the bacteria. WGS is still an important tool to monitor transmission routes of opportunistic pathogen bacteria [25, 26]. To avoid this, future missions could utilize Nanopore sequencing directly in microgravity as well as additional function and taxonomic classification methods [26, 54], and then leverage the above detailed analytic steps to gauge relevance for crew health and safety.

## Additional files

Additional file 1: **Table S1.** Genomic characteristics, single nucleotide polymorphism, single nucleotide variations, and insertion/deletions of *E. bugandensis* strains isolated from ISS. (XLSX 11 kb)

Additional file 2: **Table S2.** Plasmid gene content of ISS strains. (XLSX 17 kb)

Additional file 3: **Table S3.** Detailed function(s) of all the AMR genes associated with 5 ISS strains. (XLSX 14 kb)

## Abbreviation

AMR: Antimicrobial resistance
ANI: Average nucleotide index
ARED: Advanced Resistive Exercise Device
dDDH: Digital DNA-DNA hybridization
GGDC: Genome-to-Genome Distance Calculator
GN: Gram-negative
ISS: International Space Station
MALDI-TOF MS: Matrix-assisted laser desorption ionization–time of flight mass spectrometry
MAR: Multiple antibiotic resistance
MDR: Multiple drug resistance
MLST: Multilocus sequence typing
NASA: National Aeronautics and Space Administration
NCBI: National Center for Biotechnology Information
WGS: Whole genome sequencing
WHC: Waste and hygiene compartment
